# {4-Bromo-2-[2-(methyl­amino­)ethyl­iminometh­yl]phenolato}thio­cyanato­copper(II)

**DOI:** 10.1107/S1600536808016589

**Published:** 2008-06-07

**Authors:** Jun-Ying Ma

**Affiliations:** a Chemical Engineering and Pharmaceutics College, Henan University of Science and Technology, Luoyang, Henan 471003, People’s Republic of China, and Department of Chemistry, Pingdingshan University, Henan 467000, People’s Republic of China

## Abstract

In the title mononuclear copper(II) complex, [Cu(C_10_H_12_BrN_2_O)(NCS)], the Cu^II^ ion is coordinated by two N atoms and one O atom from a Schiff base ligand, and by one N atom from a thio­cyanate anion, giving a square-planar geometry. In the crystal structure, symmetry-related mol­ecules are linked by an N—H⋯S hydrogen bond.

## Related literature

For related literature, see: Diao & Li (2007 ▶); Diao *et al.* (2007 ▶); Ma *et al.* (2005 ▶); Ma, Gu *et al.* (2006 ▶); Ma, Lv *et al.* (2006 ▶); Ma, Wu *et al.* (2006 ▶); Wei *et al.* (2007 ▶).
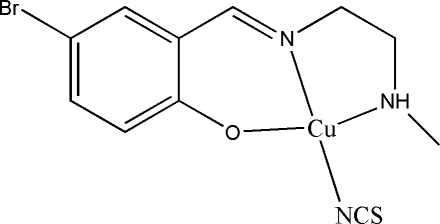


## Experimental

### 

#### Crystal data

[Cu(C_10_H_12_BrN_2_O)(NCS)]
*M* *_r_* = 377.75
Monoclinic, 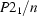

*a* = 5.952 (3) Å
*b* = 19.660 (3) Å
*c* = 12.718 (2) Å
β = 94.331 (3)°
*V* = 1484.0 (8) Å^3^
*Z* = 4
Mo *K*α radiation
μ = 4.30 mm^−1^
*T* = 298 (2) K
0.32 × 0.32 × 0.31 mm

#### Data collection

Bruker SMART CCD area-detector diffractometer
Absorption correction: multi-scan (*SADABS*; Sheldrick, 1996 ▶) *T*_min_ = 0.259, *T*_max_ = 0.267
11246 measured reflections
2997 independent reflections
1875 reflections with *I* > 2σ(*I*)
*R*_int_ = 0.071

#### Refinement

*R*[*F* ^2^ > 2σ(*F* ^2^)] = 0.076
*wR*(*F* ^2^) = 0.180
*S* = 1.11
2997 reflections
164 parameters
H-atom parameters constrained
Δρ_max_ = 1.68 e Å^−3^
Δρ_min_ = −0.84 e Å^−3^

### 

Data collection: *SMART* (Bruker, 1998 ▶); cell refinement: *SAINT* (Bruker, 1998 ▶); data reduction: *SAINT*; program(s) used to solve structure: *SHELXS97* (Sheldrick, 2008 ▶); program(s) used to refine structure: *SHELXL97* (Sheldrick, 2008 ▶); molecular graphics: *SHELXTL* (Sheldrick, 2008 ▶); software used to prepare material for publication: *SHELXTL*.

## Supplementary Material

Crystal structure: contains datablocks global, I. DOI: 10.1107/S1600536808016589/su2051sup1.cif

Structure factors: contains datablocks I. DOI: 10.1107/S1600536808016589/su2051Isup2.hkl

Additional supplementary materials:  crystallographic information; 3D view; checkCIF report

## Figures and Tables

**Table 1:** Hydrogen-bond geometry (Å, °)

*D*—H⋯*A*	*D*—H	H⋯*A*	*D*⋯*A*	*D*—H⋯*A*
N2—H2*A*⋯S1^i^	0.91	2.76	3.635 (9)	162

Symmetry code: (i) 
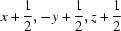
.

**Fig. 1.**
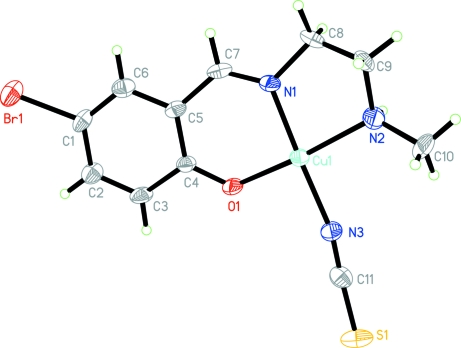
The molecular structure of compound (I), showing the atomic numbering scheme and the displacement ellipsoids drawn at the 30% probability level.

## supplementary crystallographic information

### Comment

Recently, we have reported on teh crystal structure alanyses of some metal complexes derived from Schiff base ligands (Ma, Lv *et al.*, 2006; Ma, Gu *et al.*, 2006; Ma, Wu *et al.*, 2006; Ma *et al.*, 2005). As part of a further investigation of the structures of such complexes, the title mononuclear copper(II) complex, (I), is reported on here.

In complex (I), the Cu atom is coordinated by two nitrogen atoms and one oxygen atom from a Schiff base ligand, and by one nitrogen atom from a thiocyanate anion, giving a square planar geometry (Fig. 1). All the bond lengths and angles related to the Cu atom in the complex are within normal ranges, and comparable to the values observed in other similar copper(II) complexes (Wei *et al.*, 2007; Diao *et al.*, 2007; Diao & Li, 2007). The four coordinating atoms around the Cu centre are approximately coplanar, giving a square-planar geometry with an average deviation of 0.090 (5) Å; the Cu atom lies 0.095 (2) Å above this plane. The C8—C9—N2—C10 torsion angle is 1.5 (3)°.

In the crystal structure of compound (I) symmetry related molecules are linked by an N—H···S hydrogen bond (Table 1).

### Experimental

*N*-Methylethane-1,2-diamine (0.5 mmol, 37.0 mg) and 5-bromosalicylaldehyde (0.5 mmol, 100.5 mg) were dissolved in methanol (30 ml). The mixture was stirred for 1 h to obtain a clear yellow solution. To this solution was added with stirring a methanol solution (20 ml) of copper(II) acetate (0.5 mmol, 99.6 mg) and a methanol solution (10 ml) of ammonium thiocyanate (0.5 mmol, 38.0 mg). After keeping the resulting solution in air for a few days, blue block-shaped crystals were formed.

### Refinement

All the H atoms were placed in geometrically idealized positions and constrained to ride on their parent atoms with C—H = 0.93 - 0.97 Å, with *U*_iso_(H) = 1.2 or 1.5*U*_eq_(C). An unassigned maximum residual density (1.68 Å^3^) was observed 1.03 Å from Br1, which is due to the tail-effects of the heavy atom Br1. The structure contains solvent accessible voids of 119 Å^3^, which may perhaps accommodate a partially occupied solvent molecule.

### Crystal data

**Table:**

[Cu(C_10_H_12_BrN_2_O)(NCS)]	*F*_000_ = 748
*M**_r_* = 377.75	*D*_x_ = 1.691 Mg m^−^^3^
Monoclinic, *P*2_1_/*n*	Mo *K*α radiation λ = 0.71073 Å
Hall symbol: -P 2yn	Cell parameters from 1708 reflections
*a* = 5.952 (3) Å	θ = 2.5–25.3º
*b* = 19.660 (3) Å	µ = 4.30 mm^−^^1^
*c* = 12.718 (2) Å	*T* = 298 (2) K
β = 94.331 (3)º	Block, blue
*V* = 1484.0 (8) Å^3^	0.32 × 0.32 × 0.31 mm
*Z* = 4	

### Data collection

**Table:**

Bruker SMART CCD area-detector diffractometer	2997 independent reflections
Radiation source: fine-focus sealed tube	1875 reflections with *I* > 2σ(*I*)
Monochromator: graphite	*R*_int_ = 0.071
*T* = 298(2) K	θ_max_ = 26.5º
ω scan	θ_min_ = 2.6º
Absorption correction: multi-scan(SADABS; Sheldrick, 1996)	*h* = −7→7
*T*_min_ = 0.259, *T*_max_ = 0.267	*k* = −24→24
11246 measured reflections	*l* = −15→15

### Refinement

**Table:**

Refinement on *F*^2^	Secondary atom site location: difference Fourier map
Least-squares matrix: full	Hydrogen site location: inferred from neighbouring sites
*R*[*F*^2^ > 2σ(*F*^2^)] = 0.076	H-atom parameters constrained
*wR*(*F*^2^) = 0.181	*w* = 1/[σ^2^(*F*_o_^2^) + (0.0672*P*)^2^ + 4.7203*P*] where *P* = (*F*_o_^2^ + 2*F*_c_^2^)/3
*S* = 1.11	(Δ/σ)_max_ < 0.001
2997 reflections	Δρ_max_ = 1.68 e Å^−^^3^
164 parameters	Δρ_min_ = −0.84 e Å^−^^3^
Primary atom site location: structure-invariant direct methods	Extinction correction: none

### Special details

**Table:**

Geometry. All e.s.d.'s (except the e.s.d. in the dihedral angle between two l.s. planes) are estimated using the full covariance matrix. The cell e.s.d.'s are taken into account individually in the estimation of e.s.d.'s in distances, angles and torsion angles; correlations between e.s.d.'s in cell parameters are only used when they are defined by crystal symmetry. An approximate (isotropic) treatment of cell e.s.d.'s is used for estimating e.s.d.'s involving l.s. planes.
Refinement. Refinement of *F*^2^ against ALL reflections. The weighted *R*-factor *wR* and goodness of fit *S* are based on *F*^2^, conventional *R*-factors *R* are based on *F*, with *F* set to zero for negative *F*^2^. The threshold expression of *F*^2^ > σ(*F*^2^) is used only for calculating *R*-factors(gt) *etc*. and is not relevant to the choice of reflections for refinement. *R*-factors based on *F*^2^ are statistically about twice as large as those based on *F*, and *R*- factors based on ALL data will be even larger.

### Fractional atomic coordinates and isotropic or equivalent isotropic displacement parameters (Å^2^)

**Table:**

	*x*	*y*	*z*	*U*_iso_*/*U*_eq_	
Cu1	0.77313 (15)	0.20764 (5)	0.04645 (8)	0.0407 (3)	
N1	1.0514 (10)	0.1635 (3)	0.0963 (5)	0.0392 (15)	
N2	0.8291 (12)	0.2634 (4)	0.1796 (7)	0.058 (2)	
H2A	0.7645	0.2393	0.2304	0.070*	
N3	0.4927 (12)	0.2539 (4)	0.0002 (6)	0.053 (2)	
O1	0.7104 (8)	0.1410 (3)	−0.0588 (4)	0.0460 (14)	
S1	0.0695 (4)	0.29376 (15)	−0.0863 (2)	0.0664 (8)	
Br1	1.31654 (16)	−0.06412 (5)	−0.23999 (9)	0.0633 (4)	
C1	1.1328 (13)	0.0028 (4)	−0.1792 (7)	0.044 (2)	
C2	0.9203 (14)	0.0131 (4)	−0.2253 (8)	0.049 (2)	
H2	0.8712	−0.0121	−0.2845	0.059*	
C3	0.7799 (13)	0.0596 (4)	−0.1860 (7)	0.047 (2)	
H3	0.6360	0.0657	−0.2184	0.057*	
C4	0.8518 (13)	0.0994 (4)	−0.0946 (6)	0.0367 (18)	
C5	1.0688 (12)	0.0860 (4)	−0.0492 (6)	0.0369 (18)	
C6	1.2068 (14)	0.0376 (4)	−0.0913 (7)	0.045 (2)	
H6	1.3497	0.0291	−0.0592	0.054*	
C7	1.1533 (13)	0.1183 (4)	0.0480 (7)	0.042 (2)	
H7	1.2941	0.1047	0.0773	0.051*	
C8	1.1509 (15)	0.1902 (5)	0.1982 (7)	0.052 (2)	
H8A	1.3140	0.1889	0.1997	0.062*	
H8B	1.1038	0.1625	0.2557	0.062*	
C9	1.0734 (14)	0.2612 (5)	0.2099 (7)	0.051 (2)	
H9A	1.1028	0.2760	0.2824	0.061*	
H9B	1.1531	0.2912	0.1650	0.061*	
C10	0.7370 (17)	0.3305 (6)	0.1855 (10)	0.083 (4)	
H10A	0.8001	0.3526	0.2482	0.125*	
H10B	0.5763	0.3276	0.1874	0.125*	
H10C	0.7728	0.3562	0.1248	0.125*	
C11	0.3146 (15)	0.2696 (4)	−0.0378 (7)	0.048 (2)	

### Atomic displacement parameters (Å^2^)

**Table:**

	*U*^11^	*U*^22^	*U*^33^	*U*^12^	*U*^13^	*U*^23^
Cu1	0.0271 (5)	0.0511 (6)	0.0429 (6)	0.0011 (4)	−0.0028 (4)	−0.0086 (5)
N1	0.028 (3)	0.047 (4)	0.041 (4)	−0.004 (3)	−0.006 (3)	−0.001 (3)
N2	0.040 (4)	0.062 (5)	0.070 (5)	−0.001 (4)	−0.005 (4)	−0.022 (4)
N3	0.030 (4)	0.069 (5)	0.058 (5)	0.008 (3)	−0.006 (3)	−0.015 (4)
O1	0.025 (3)	0.055 (4)	0.056 (4)	0.006 (3)	−0.009 (2)	−0.012 (3)
S1	0.0412 (13)	0.0813 (18)	0.0744 (18)	0.0000 (13)	−0.0115 (12)	0.0316 (15)
Br1	0.0480 (6)	0.0520 (6)	0.0910 (8)	0.0037 (4)	0.0131 (5)	−0.0149 (5)
C1	0.036 (4)	0.030 (4)	0.068 (6)	0.003 (3)	0.014 (4)	−0.002 (4)
C2	0.035 (5)	0.045 (5)	0.066 (6)	−0.005 (4)	0.001 (4)	−0.009 (4)
C3	0.029 (4)	0.047 (5)	0.064 (6)	0.000 (4)	−0.014 (4)	−0.007 (4)
C4	0.031 (4)	0.033 (4)	0.044 (5)	0.001 (3)	−0.006 (3)	−0.002 (4)
C5	0.029 (4)	0.040 (4)	0.041 (5)	0.004 (3)	0.004 (3)	0.013 (4)
C6	0.042 (5)	0.037 (4)	0.057 (5)	0.000 (4)	−0.002 (4)	0.007 (4)
C7	0.028 (4)	0.043 (5)	0.055 (5)	0.003 (4)	−0.007 (4)	0.015 (4)
C8	0.048 (5)	0.065 (6)	0.039 (5)	−0.011 (4)	−0.017 (4)	−0.003 (4)
C9	0.039 (5)	0.075 (6)	0.038 (5)	−0.006 (4)	−0.002 (4)	−0.022 (5)
C10	0.051 (6)	0.092 (8)	0.106 (9)	0.038 (6)	−0.005 (6)	−0.039 (7)
C11	0.042 (5)	0.051 (5)	0.050 (5)	−0.015 (4)	−0.001 (4)	−0.004 (4)

### Geometric parameters (Å, °)

**Table:**

Cu1—O1	1.890 (5)	C2—H2	0.9300
Cu1—N1	1.934 (6)	C3—C4	1.439 (11)
Cu1—N3	1.952 (7)	C3—H3	0.9300
Cu1—N2	2.024 (7)	C4—C5	1.399 (10)
N1—C7	1.262 (10)	C5—C6	1.390 (11)
N1—C8	1.479 (10)	C5—C7	1.446 (12)
N2—C10	1.432 (12)	C6—H6	0.9300
N2—C9	1.476 (11)	C7—H7	0.9300
N2—H2A	0.9100	C8—C9	1.481 (12)
N3—C11	1.172 (11)	C8—H8A	0.9700
O1—C4	1.282 (9)	C8—H8B	0.9700
S1—C11	1.612 (10)	C9—H9A	0.9700
Br1—C1	1.911 (8)	C9—H9B	0.9700
C1—C6	1.356 (12)	C10—H10A	0.9600
C1—C2	1.368 (12)	C10—H10B	0.9600
C2—C3	1.358 (12)	C10—H10C	0.9600
			
O1—Cu1—N1	92.3 (3)	C6—C5—C4	121.6 (8)
O1—Cu1—N3	89.5 (3)	C6—C5—C7	116.9 (7)
N1—Cu1—N3	178.1 (3)	C4—C5—C7	121.4 (7)
O1—Cu1—N2	168.3 (3)	C1—C6—C5	119.9 (8)
N1—Cu1—N2	83.4 (3)	C1—C6—H6	120.1
N3—Cu1—N2	94.7 (3)	C5—C6—H6	120.1
C7—N1—C8	120.0 (7)	N1—C7—C5	125.2 (7)
C7—N1—Cu1	126.0 (5)	N1—C7—H7	117.4
C8—N1—Cu1	113.9 (5)	C5—C7—H7	117.4
C10—N2—C9	112.8 (8)	N1—C8—C9	108.4 (7)
C10—N2—Cu1	120.2 (7)	N1—C8—H8A	110.0
C9—N2—Cu1	107.4 (5)	C9—C8—H8A	110.0
C10—N2—H2A	105.0	N1—C8—H8B	110.0
C9—N2—H2A	105.0	C9—C8—H8B	110.0
Cu1—N2—H2A	105.0	H8A—C8—H8B	108.4
C11—N3—Cu1	166.5 (7)	N2—C9—C8	108.1 (7)
C4—O1—Cu1	126.5 (5)	N2—C9—H9A	110.1
C6—C1—C2	120.7 (8)	C8—C9—H9A	110.1
C6—C1—Br1	121.4 (6)	N2—C9—H9B	110.1
C2—C1—Br1	117.9 (7)	C8—C9—H9B	110.1
C3—C2—C1	121.1 (8)	H9A—C9—H9B	108.4
C3—C2—H2	119.4	N2—C10—H10A	109.5
C1—C2—H2	119.4	N2—C10—H10B	109.5
C2—C3—C4	120.6 (7)	H10A—C10—H10B	109.5
C2—C3—H3	119.7	N2—C10—H10C	109.5
C4—C3—H3	119.7	H10A—C10—H10C	109.5
O1—C4—C5	125.7 (7)	H10B—C10—H10C	109.5
O1—C4—C3	118.1 (7)	N3—C11—S1	177.5 (9)
C5—C4—C3	116.1 (7)		

### Hydrogen-bond geometry (Å, °)

**Table:**

*D*—H···*A*	*D*—H	H···*A*	*D*···*A*	*D*—H···*A*
N2—H2A···S1^i^	0.91	2.76	3.635 (9)	162

Symmetry codes: (i) *x*+1/2, −*y*+1/2, *z*+1/2.

## References

[bb1] Bruker (1998). *SMART* and *SAINT* Bruker AXS Inc., Madison, Wisconsin, USA.

[bb2] Diao, Y.-P. & Li, K. (2007). *Acta Cryst.* E**63**, m2496–m2497.

[bb3] Diao, Y.-P., Shu, X.-H., Zhang, B.-J., Zhen, Y.-H. & Kang, T.-G. (2007). *Acta Cryst.* E**63**, m1816.

[bb4] Ma, J.-Y., Gu, S.-H., Guo, J.-W., Lv, B.-L. & Yin, W.-P. (2006). *Acta Cryst.* E**62**, m1437–m1438.

[bb5] Ma, J.-Y., Lv, B.-L., Gu, S.-H., Guo, J.-W. & Yin, W.-P. (2006). *Acta Cryst.* E**62**, m1322–m1323.

[bb6] Ma, J.-Y., Wu, T.-X., She, X.-G. & Pan, X.-F. (2005). *Acta Cryst.* E**61**, m695–m696.

[bb7] Ma, J.-Y., Wu, T.-X., She, X.-G. & Pan, X.-F. (2006). *Z. Kristallogr. New Cryst. Struct.* **221**, 53–54.

[bb8] Sheldrick, G. M. (1996). *SADABS* University of Göttingen, Germany.

[bb9] Sheldrick, G. M. (2008). *Acta Cryst.* A**64**, 112–122. 10.1107/S0108767307043930 18156677

[bb10] Wei, Y.-J., Wang, F.-W. & Zhu, Q.-Y. (2007). *Acta Cryst.* E**63**, m2629.

